# Distinguishing Primary Prevention From Secondary Prevention Implantable Cardioverter Defibrillators Using Administrative Health and Cardiac Device Registry Data

**DOI:** 10.1016/j.cjco.2024.02.003

**Published:** 2024-03-07

**Authors:** Isaac Robinson, Daniel Daly-Grafstein, Mayesha Khan, Andrew D. Krahn, Nathaniel M. Hawkins, Jeffrey R. Brubacher, John A. Staples

**Affiliations:** a Department of Medicine, University of British Columbia, Vancouver, British Columbia, Canada; b Center for Cardiovascular Innovation, Division of Cardiology, University of British Columbia, Vancouver, British Columbia, Canada; c Department of Emergency Medicine, University of British Columbia, Vancouver, British Columbia, Canada; d Division of General Internal Medicine, Department of Medicine, University of British Columbia, Vancouver, British Columbia, Canada; e Centre for Clinical Epidemiology & Evaluation (C2E2), Vancouver, British Columbia, Canada

## Abstract

**Background:**

Administrative health data and cardiac device registries can be used to empirically evaluate outcomes and costs after implantable cardioverter defibrillator (ICD) implantation. These datasets often have incomplete information on the indication for implantation (primary vs secondary prevention of sudden cardiac death).

**Methods:**

We used 16 years of population-based cardiac device registry and administrative health data from British Columbia, Canada, to derive and internally validate statistical models that predict the likely indication for ICD implantation. We used chart review data as the reference standard for ICD indication in the Cardiac Device Registry database (CDR; 2004-2012 [Cardiac Services BC]) and nonmissing indication as the reference standard in the Heart Information System registry database (HEARTis; 2013-2019 [Cardiac Services BC]). We created 3 logistic regression prediction models in each database: one using only registry data, one using only administrative data, and one using both registry and administrative data. We assessed the predictive performance of each model using standard metrics after optimism correction with 200 bootstrap resamples.

**Results:**

Models that used registry data alone demonstrated excellent predictive performance (sensitivity ≥ 89%; specificity ≥ 87%). Models that used only administrative data performed well (sensitivity ≥ 84%; specificity ≥ 70%). Models that used both registry and administrative data showed modest gains over those that used registry data alone (sensitivity ≥ 90%; specificity ≥ 89%).

**Conclusions:**

Administrative health data and cardiac device registry data can distinguish secondary prevention ICDs from primary prevention ICDs with acceptable sensitivity and specificity. Imputation of missing ICD indication might make these data resources more useful for research and health system monitoring.

## Résumé

**Contexte:**

Les données administratives de santé et les registres des dispositifs cardiaques peuvent être utilisés pour évaluer de manière empirique les résultats et les coûts associés aux défibrillateurs cardioverteurs implantables (DCI). Or, ces ensembles de données comportent souvent des informations incomplètes sur l’indication de l’implant (prévention primaire ou secondaire de la mort subite d’origine cardiaque).

**Méthodologie:**

Nous avons analysé 16 ans de données provenant du registre populationnel des dispositifs cardiaques et des données administratives de santé de la Colombie-Britannique, au Canada, pour alimenter des modèles statistiques prédisant l’indication probable de l’implant d’un DCI et pour effectuer une validation interne de ces modèles. Nous avons utilisé les données de la revue des dossiers médicaux comme norme de référence de l’indication des DCI dans le registre des dispositifs cardiaques (*Cardiac Device Registry*; 2004-2012 [Cardiac Services BC]) et les indications consignées comme norme de référence dans la banque de données *Heart Information System* (HEARTis; 2013-2019 [Cardiac Services BC]). Nous avons créé 3 modèles prédictifs par régression logistique dans chaque base de données : une utilisant seulement les données du registre, une utilisant seulement les données administratives et une utilisant les deux types de données. Nous avons évalué la performance de chaque modèle en matière de prédiction à l’aide de mesures normalisées, après correction pour l’optimisme de l’erreur à l’aide de 200 nouveaux échantillons obtenus par la méthode *bootstrap*.

**Résultats:**

Les modèles utilisant seulement les données du registre avaient une excellente performance prédictive (sensibilité ≥ 89 %; spécificité ≥ 87 %). Les modèles qui n’utilisaient que les données administratives donnaient quant à eux de bons résultats (sensibilité ≥ 84 %; spécificité ≥ 70 %). Enfin, les modèles qui utilisaient les données administratives et les données du registre ont donné des gains modestes par rapport aux modèles qui n’utilisaient que les données du registre (sensibilité ≥ 90 %; spécificité ≥ 89 %).

**Conclusions:**

Les données administratives de santé et les données des registres de dispositifs cardiaques permettent de distinguer les DCI implantés en prévention secondaire des DCI implantés en prévention primaire avec une sensibilité et une spécificité acceptables. Dans les cas où elle est absente, l’attribution d’une indication pour les DCI pourrait donc rendre ces ressources plus utiles pour la recherche et la surveillance du système de santé.

Every year, over 150,000 patients in the US and 7000 patients in Canada receive an implantable cardioverter defibrillator (ICD) for the prevention of sudden cardiac death (SCD).^1^^,^^2^ Despite the life-saving benefits of ICDs, up to 9% of patients experience a complication in the first 16 months after implantation,^3^ and approximately 1 in 4 transvenous ICD leads have a mechanical complication within 10 years.^4^ ICDs also are expensive, with previous estimates suggesting a cost of up to US∖$70,200 per quality-adjusted life-year gained.^5^

Two main sources of routinely collected health data could improve our understanding of the real-world benefits, risks, and cost-effectiveness of ICDs.^6^ *Cardiac device registries* include granular clinical information from the time of implantation but usually lack data on external costs and long-term outcomes.^7^ *Administrative health data* are collected for billing and healthcare insurance purposes and typically lack granular clinical information. However, they often include data on most medical services used by a large population over a long period of time, making it possible to select control subjects, estimate overall healthcare system costs, and evaluate longer-term outcomes (an important feature when studying preventative treatments such as ICDs).^6^ Compared to randomized controlled trials, retrospective studies using routinely collected health data can be faster, cheaper, easier to perform, and potentially less prone to commercial conflicts of interest.^6^^,^^8^ These advantages are valuable because assessments of effectiveness need to be revised periodically as ICD technology improves (eg, new device programming can affect shock incidence; different models of transvenous leads can affect complication rates).^4^^,^^9^ These features also make routinely collected health data useful for health system surveillance, including assessment of uptake and evaluation of disparities in access to ICDs.^10^^,^^11^

Establishing the indication for ICD implantation in routinely collected health data is particularly important to allow reassessment of the benefits and risks of primary prevention ICDs.^8^ Heart failure patients who would be candidates to receive primary prevention ICDs have seen a decline in SCD risk in recent decades because of improved medical therapy and larger competing risks for mortality in an older and more comorbid population.^8^^,^^12^^,^^13^ Evidence from older landmark randomized control trials may therefore no longer reflect current clinical reality. Unfortunately, the indication for ICD implantation is missing from routinely collected health data in many countries.^6^^,^^10^^,^^11^^,^^14^, ^15^, ^16^ Indication for ICD implantation can be missing from cardiac device registries because data entry into the registry may not be compulsory, physician documentation may be incomplete, and funding for data collection may be inconsistent.^14^^,^^16^, ^17^, ^18^ Indication can be missing from administrative health data because these details are often irrelevant for billing purposes.^6^ These data gaps are a major barrier to research and health system surveillance.

Accordingly, we used 16 years of population-based administrative health and cardiac device registry data to derive and internally validate statistical models to predict the indication for ICD implantation.

## Methods

### Setting

Our study was set in British Columbia (BC), Canada. BC's 5.4 million residents are universally insured for medically necessary health services including ICD implantation.^19^ ICD implantations are performed at only 5 specialized hospitals in the province.

### Registry data

We obtained population-based cardiac device registry data from Cardiac Services BC (CSBC). CSBC cardiac device registries include data on all cardiac devices implanted throughout the province. Between 1995 and 2013, CSBC collected these data in their Cardiac Device Registry (CDR). The CDR originally did not capture the indication for ICD implantation, but Hawkins et al. later performed a retrospective clinical record review study to establish indication for ICD implantations occurring between January 2003 and March 2012.^14^ We used data from this chart review as the reference standard for ICD indication in the CDR (although only data from 2004 to 2012 were available to us). Starting on October 11, 2013, CSBC switched to a new data collection form and a new registry database (the Heart Information System [HEARTis]). HEARTis has a specific field where ICD indication can be specified, but entries for this field are sometimes missing. We used recorded (nonmissing) indication as the reference standard in HEARTis. The current study used registry data from these sources for ICDs implanted between January 1, 2004 and October 31, 2019.

### Administrative data

We used unique provincial Personal Health Numbers (PHNs) to deterministically link cardiac device registry data to population-based, individual-level administrative health data that includes information on hospitalizations, physician fee-for-service claims, and all outpatient prescription medications filled at any community pharmacy in BC (Supplemental Table S1**)**.^20^ We identified ICD implantations in the administrative data by finding the hospital episode-of-care record that corresponded to the implantation date from the cardiac device registry and included a Canadian Classification of Health Intervention code denoting ICD implantation (ie, 1HZ53GRFS, 1HZ53GRFU, 1HZ53HNFS, 1HZ53LAFS, 1HZ53LAFU, 1HZ53SYFS, 1HZ53SYFU, 1HZ53HAFS).

We deemed comorbidities to be present in the administrative data when, over a 1-year look-back interval, relevant diagnostic codes were found in any of the 25 diagnosis fields for ≥ 1 hospitalizations or in any of the 5 diagnosis fields for ≥ 2 physician visits, using diagnostic codes from the International Statistical Classification of Diseases and Related Health Problems, 10^th^ revision, Canadian version (ICD-10-CA in the Discharge Abstract Database and International Classification of Diseases, 9th revision—Clinical Modification [ICD-9-CM] in the Medical Services Plan data). We identified active prescription medication use based on the dispensation date and days supplied for all prescriptions.

### Cohort, predictors, and analysis

Our study cohort included an individual's first ICD implantation if it met the following criteria: (i) occurred between January 1, 2004 and October 31, 2019; (ii) could be identified in both the cardiac device registry and the administrative health data; (iii) had an indication for implantation recorded in the corresponding reference standard; and (iv) had a device type recorded in the cardiac device registry data (ie, single-chamber, dual-chamber, cardiac resynchronization therapy). We identified potential predictors of ICD indication based on clinical plausibility (Supplemental Tables S2 and S3).

We fit several logistic regression models that used variables from the cardiac device registry and from the administrative health data to predict whether an ICD was implanted for secondary prevention of SCD. We fit separate models in the CDR (2004-2012) and in HEARTis (2013-2019) because each database collected different variables. When a particular clinical feature was captured by both administrative and registry data and we were fitting a model using both data sources, we deemed the comorbidity or medication to be present if it was identified as present in either database. We did not perform any variable selection, because our goal was to predict indication instead of establishing causality. We compared the predicted ICD indication to the ICD indication established using the reference standard and assessed the sensitivity, specificity, positive predictive value, negative predictive value, and C-statistic (area under the receiver operating curve) of the predictive algorithm after optimism correction with 200 bootstrap resamples.^21^

### Ethics

The University of BC Clinical Research Ethics Board approved the study and waived the requirement for individual consent (H16-02043). Analysis occurred between January 2022 and September 2023, using R, version 4.0 (R Foundation, Vienna, Austria). All inferences, opinions, and conclusions drawn are those of the authors and do not reflect the opinions or policies of the Data Stewards. Research reporting adheres to the Strengthening the Reporting of Observational Studies in Epidemiology (STROBE) guidelines.

## Results

**Figure 1:**
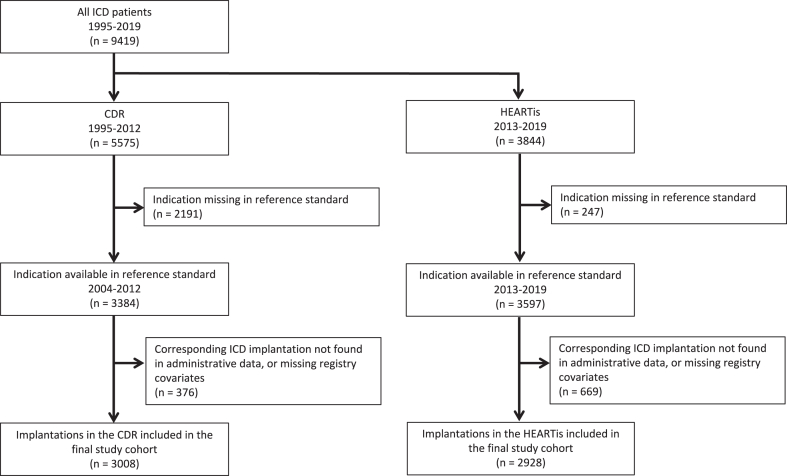
Flow diagram, beginning with all patients receiving their first implantable cardioverter defibrillator (ICD) in British Columbia between 1995 and 2019, and ending with the final study cohort. We used chart review data as the reference standard in the Cardiac Device Registry (CDR; Cardiac Services BC). We used the nonmissing indications in the Heart Information System (HEARTis; Cardiac Services BC) as the reference standard in this subset.

**Figure 2:**
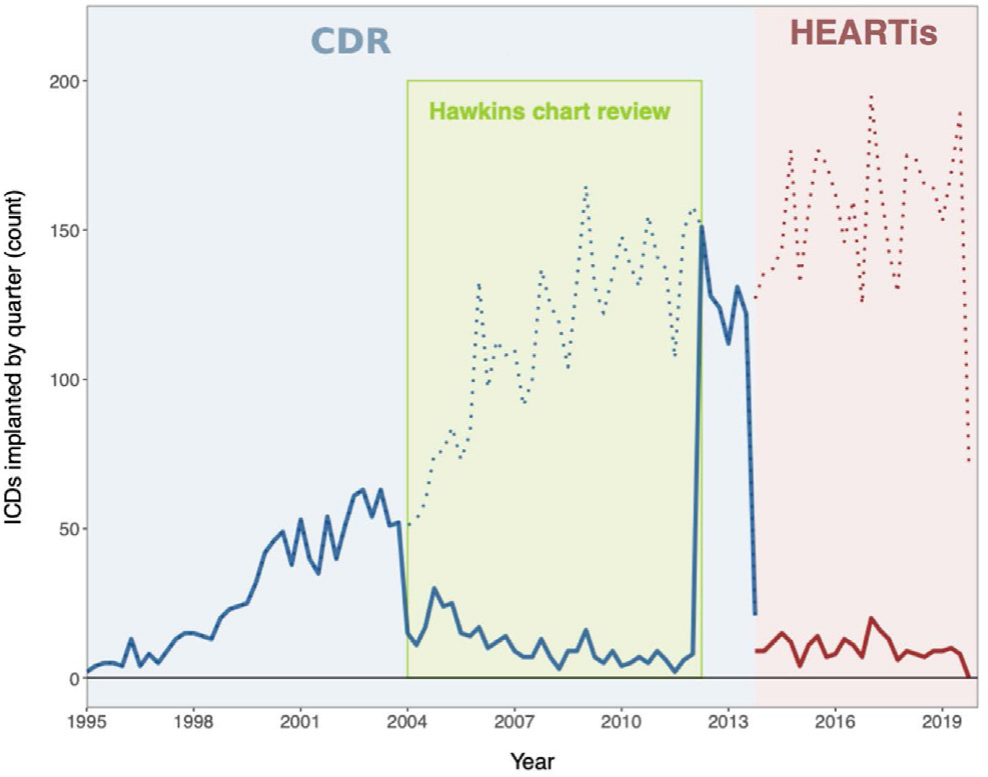
Implantable cardioverter defibrillators (ICDs) implanted and missingness of ICD indication over time. Cardiac Services BC collected data on ICD implantations in its Cardiac Device Registry (CDC) from 1995 to 2013 (**blue shading**), and then in the Heart Information System (HEARTis) database from October of 2013 until 2019 (**red shading**). Hawkins et al.^14^ performed a chart review of all ICD implantations between 2003 and 2012; however, the portion of this indication data available for the current study begins in 2004 (**green shading**). The **solid line** depicts the number of ICDs implanted per quarter with missing indication, and the **dashed line** depicts the total number of ICDs implanted per quarter. The main finding is that only a small proportion of patients have missing data on indication for implantation during the Hawkins or HEARTis period.

The final study cohort consisted of 5936 first ICD implantations (Fig. 1). The number of ICDs implanted annually increased more than 3-fold over the 16-year study interval (Fig. 2**)**. In total, 1341 of the 3008 ICDs included from the CDR (45%), and 1436 of the 2928 ICDs included from HEARTis (49%), were implanted for secondary prevention of SCD.

The final study cohort was predominantly male and had a median age of 66 years (Table 1). Baseline comorbidities and active medications were typical of individuals undergoing ICD implantation, with a majority having a history of ischemic heart disease, heart failure, and cardiac arrhythmia, and a majority having been prescribed beta-blockers and angiotensin-converting enzyme (ACE) inhibitors.

**Table 1:** Baseline characteristics

Characteristic	Entire CDR (1995–2012), n = 5575	CDR included in final cohort∗, n = 3008 (54%)	Entire HEARTis (2013–2019), n = 3844	HEARTis included in final cohort∗, n = 2928 (76%)
Demographics				
Median age, y [Q1, Q3]	65 [55.5, 72]	65.5 [57, 73]	67 [58, 74]	67 (58, 74)
Male sex	4575 (82.1)	2465 (81.9)	3026 (78.7)	2324 (79.4)
Neighbourhood income quintile				
First (lowest income)	1056 (18.9)	561 (18.7)	757 (19.7)	586 (20)
Second	1151 (20.6)	643 (21.4)	723 (18.8)	558 (19.1)
Third	1100 (19.7)	618 (20.5)	776 (20.2)	605 (20.7)
Fourth	1044 (18.7)	566 (18.8)	766 (19.9)	598 (20.4)
Fifth (highest income)	1080 (19.4)	574 (19.1)	710 (18.5)	547 (18.7)
Missing	144 (2.6)	46 (1.5)	112 (2.9)	34 (1.2)
Medical history in prior year				
CCI ≥ 2	2870 (51.5)	1646 (54.7)	2150 (55.9)	1668 (57)
≥ 1 hospitalization	5514 (98.9)	3008 (100)	3776 (98.2)	2928 (100.0)
≥ 7 physician visits	5420 (97.2)	2974 (98.9)	3717 (96.7)	2885 (98.5)
Any heart condition	5453 (97.8)	2975 (98.9)	3712 (96.6)	2885 (98.5)
Myocardial infarction	1529 (27.4)	784 (26.1)	958 (24.9)	741 (25.3)
Congestive heart failure	4079 (73.2)	2402 (79.9)	2769 (72.0)	2161 (73.8)
Peripheral vascular disease	437 (7.8)	225 (7.5)	179 (4.7)	134 (4.6)
Cerebrovascular disease	278 (5.0)	151 (5.0)	192 (5.0)	139 (4.7)
Dementia	31 (0.6)	23 (0.8)	37 (1.0)	26 (0.9)
Chronic obstructive pulmonary disease	621 (11.1)	345 (11.5)	428 (11.1)	334 (11.4)
Rheumatic disease	57 (1.0)	30 (1.0)	30 (0.8)	25 (0.9)
Peptic ulcer disease	59 (1.1)	33 (1.1)	39 (1.0)	29 (1.0)
Mild liver disease	48 (0.9)	26 (0.9)	58 (1.5)	44 (1.5)
Diabetes	1407 (25.2)	852 (28.3)	1229 (32.0)	947 (32.3)
Paraplegia and hemiplegia	33 (0.6)	17 (0.6)	16 (0.4)	9 (0.3)
Renal disease	479 (8.6)	280 (9.3)	591 (15.4)	456 (15.6)
Cancer	181 (3.2)	95 (3.2)	151 (3.9)	119 (4.1)
Moderate or severe liver disease	8 (0.1)	0 (0)	11 (0.3)	8 (0.3)
Metastatic carcinoma	16 (0.3)	≤ 5	12 (0.3)	7 (0.2)
HIV	9 (0.2)	≤ 5	12 (0.3)	11 (0.4)
Syncope	537 (9.6)	279 (9.3)	401 (10.4)	312 (10.7)
Atrial fibrillation and flutter	1367 (24.5)	736 (24.5)	1027 (26.7)	804 (27.5)
Other arrhythmias	4674 (83.8)	2461 (81.8)	2981 (77.5)	2,313 (79)
Seizure disorders	48 (0.9)	22 (0.7)	44 (1.1)	34 (1.2)
Obstructive sleep apnea and other sleep disorders	142 (2.5)	81 (2.7)	126 (3.3)	99 (3.4)
Psychiatric disorders	734 (13.2)	410 (13.6)	601 (15.6)	466 (15.9)
Alcohol misuse	161 (2.9)	87 (2.9)	113 (2.9)	82 (2.8)
Other substance misuse	121 (2.2)	51 (1.7)	79 (2.1)	62 (2.1)
Chronic ischemic heart disease	3492 (62.6)	1926 (64.0)	2252 (58.6)	1769 (60.4)
Hypertension	2404 (43.1)	1356 (45.1)	1759 (45.8)	1366 (46.7)
Unstable angina	228 (4.1)	123 (4.1)	55 (1.4)	42 (1.4)
Pacemaker	≤ 5	0 (0)	59 (1.5)	37 (1.3)
Cardiac arrest	1036 (18.6)	523 (17.4)	898 (23.4)	714 (24.4)
Ventricular tachycardia or fibrillation	3778 (67.8)	1886 (62.7)	1849 (48.1)	1456 (49.7)
Medications active at baseline				
Number of medications				
0 or 1	1428 (25.6)	653 (21.7)	1004 (26.1)	744 (25.4)
≥ 2	4147 (74.4)	2355 (78.3)	2840 (73.9)	2184 (74.6)
Loop diuretics	1859 (33.3)	1094 (36.4)	1058 (27.5)	821 (28.0)
ACEi or ARB	3193 (57.3)	1867 (62.1)	2119 (55.1)	1631 (55.7)
MRA	1287 (23.1)	788 (26.2)	1033 (26.9)	830 (28.3)
Beta-blockers	3166 (56.8)	1852 (61.6)	2180 (56.7)	1688 (57.7)
Nitroglycerin	558 (10.0)	306 (10.2)	234 (6.1)	180 (6.1)
Hydralazine	83 (1.5)	50 (1.7)	63 (1.6)	46 (1.6)
Antihypertensives	4172 (74.8)	2363 (78.6)	2751 (71.6)	2116 (72.3)
Digoxin	883 (15.8)	489 (16.3)	190 (4.9)	143 (4.9)
Calcium-channel blockers	511 (9.2)	276 (9.2)	326 (8.5)	229 (7.8)
Anti-arrhythmics	537 (9.6)	256 (8.5)	155 (4.0)	123 (4.2)
Statins	2395 (43.0)	1448 (48.1)	1790 (46.6)	1376 (47.0)
Antiplatelets	769 (13.8)	461 (15.3)	685 (17.8)	542 (18.5)
Anticoagulants	1153 (20.7)	660 (21.9)	863 (22.5)	650 (22.2)
Insulin	241 (4.3)	143 (4.8)	201 (5.2)	150 (5.1)
Oral hypoglycemics	743 (13.3)	463 (15.4)	667 (17.4)	504 (17.2)
Opioids	319 (5.7)	191 (6.3)	254 (6.6)	201 (6.9)
Benzodiazepines	643 (11.5)	357 (11.9)	307 (8.0)	243 (8.3)

Baseline characteristics for the study cohort from both cardiac device registries and administrative health data. Values are n (%), unless otherwise indicated.

ACEi, angiotensin-converting enzyme inhibitor; ARB, angiotensin receptor blocker; CCI, Charlson Comorbidity Index; CDR, Cardiac Device Registry [Cardiac Services BC]; HEARTis, Heart Information System [Cardiac Services BC]; HIV, human immunodeficiency virus; MRA, mineralocorticoid receptor antagonist; Q, quartile.

∗ See text for inclusion criteria. Myocardial infarction, other substance misuse, chronic ischemic heart disease, hypertension, unstable angina, and pacemaker were considered present if ≥ 1 entries were present in the Discharge Abstract Database (DAD) data or ≥ 2 entries were present in the Medical Services Plan (MSP) data.

Predictive models that used both registry and administrative data exhibited excellent ability to predict which ICDs were implanted for secondary prevention (sensitivity ≥ 90%, specificity ≥ 89%, positive predictive value ≥ 91%, and negative predictive value ≥ 87%, in both the CDR and HEARTis; Table 2). Predictive models that used registry data alone also exhibited excellent performance (sensitivity ≥ 89%, specificity ≥ 87%, positive predictive value ≥ 87%, and negative predictive value ≥ 86% in both the CDR and HEARTis). Predictive models based on administrative data alone exhibited very good performance, particularly for the more recently implanted ICDs in the HEARTis database (sensitivity ≥ 84%, specificity ≥ 70%, positive predictive value ≥ 78%, and negative predictive value 78% in both the CDR and HEARTis).

**Table 2:** Predictive model performance using registry data, administrative data, or both

Measure of predictive model performance (optimism-corrected)	CDR (2004–2012), n = 3008			HEARTis (201–2019), n = 2928			Entire data set (2004–2019), n = 5936
	Both	Registry only	Admin only	Both	Registry only	Admin only	Basic admin variables only∗
Sensitivity	0.90	0.89	0.84	0.92	0.89	0.90	0.82
Specificity	0.89	0.88	0.70	0.91	0.87	0.89	0.71
Positive predictive value	0.91	0.90	0.78	0.92	0.87	0.89	0.76
Negative predictive value	0.87	0.86	0.78	0.92	0.89	0.90	0.78
C-statistic	0.97	0.96	0.86	0.97	0.95	0.96	0.85

Table displays performance metrics for models using only administrative (admin) health data, only cardiac device registry data, and models combining both. Performance metrics were corrected for optimism (the inflation of model performance metrics when evaluated on the same data they are trained on) with 200 bootstraps on the entire training set. The Cardiac Device Registry (CDR; Cardiac Services BC) and Heart Information System (HEARTis; Cardiac Services BC) subsets included in this study consists of only implantable cardioverter defibrillator cases with nonmissing indication that also have corresponding records in admin data. The C-statistic measures the goodness-of-fit of the logistic regression. Table shows excellent model performance in each subset of data.

∗ This model used only basic administrative variables, including age, sex, features of the index hospital visit for ICD implantation (urgency of admission, length of stay ≥ 3 days), and the presence of cardiac arrest, ventricular arrhythmia, and heart failure on the index implantation hospital visit record.

Using registry data alone for both the CDR and HEARTis, history of any of cardiac arrest, ventricular fibrillation, or Brugada syndrome was an extremely strong predictor of secondary prevention ICD, increasing the likelihood by about 2500-fold in the CDR, and deterministically predicting secondary prevention in HEARTis. Elective (rather than “urgent/emergency”) implantation reduced the likelihood of secondary prevention ICD by about half. Left ventricular ejection fraction and New York Heart Association class were not available from the CDR but were only modest predictors of ICD indication in HEARTis. Other predictors were highly plausible, supporting the face validity of our approach (Supplemental Tables S4 and S5).

Using administrative data alone for both the CDR and HEARTis, the identification of specific comorbidities during the index hospital visit for ICD implantation changed the likelihood of a secondary prevention ICD: A history of cardiac arrest increased the likelihood by almost 10-fold; a history of ventricular arrhythmia more than doubled the likelihood; and a history of heart failure reduced the likelihood by about 60% (Supplemental Table S6). The specific medications that were active at implantation date had a modest influence on the likelihood of secondary prevention ICD.

Based on our findings, we also evaluated a simplified predictive model based on administrative data alone that included only age, sex, features of the index hospital visit for ICD implantation (urgency of admission, length of stay ≥ 3 days), and an index implantation hospital visit record that notes current or prior cardiac arrest, ventricular arrhythmia, or heart failure. Using pooled data from the final study cohort, this simplified predictive model exhibited adequate performance for the prediction of secondary prevention ICDs (Table 2; sensitivity 82%, specificity 71%, positive predictive value 76%, negative predictive value 78%, and C-statistic 0.85).

## Discussion

Using 16 years of retrospective population-based data for 5936 ICD implantations, we found that cardiac device registry and administrative health data could be used to predict which ICDs were implanted for secondary prevention of SCD. We found that predictive models based on a combination of registry data and administrative health data demonstrated excellent predictive performance, models based on registry data alone demonstrated excellent predictive performance, and models based on administrative health data alone demonstrated acceptable performance. These findings have potential utility to researchers (who may wish to evaluate ICD uptake, benefits, risks, and cost-effectiveness), to administrators (who may wish to monitor healthcare system performance), and to clinicians (who may wish to compare practice patterns with peers). To help with these tasks, we provide data on the degree to which specific demographic, clinical, and procedural variables predict that an ICD was implanted for secondary prevention of SCD (Supplemental Tables S4-S8). We note that similar variables are frequently available in other cardiac device registries, highlighting the potential generalizability of our work.^22^, ^23^, ^24^, ^25^

Prior studies would have benefitted from a validated method for establishing the indication for ICD implantation.^6^^,^^10^^,^^11^^,^^15^^,^^16^ A pioneering 2013 Italian study used administrative health data to examine the real-world impacts and costs of ICD treatment, but the analysis was limited by a lack of data on indication.^6^ Cost-benefit analyses that ignore ICD indication may misinform clinicians on the extent to which the treatment is beneficial. A retrospective cohort of 14,230 patients in the US examined ICD shock recurrence in a remote monitoring registry, relying on recorded reasons for device implantation, such as “ventricular tachycardia” to indicate a secondary prevention ICD, or “congestive heart failure” to indicate a primary prevention ICD.^15^ However, 61% of the cohort had to be excluded because the markers for inferring primary or secondary prevention were not available. A nationwide assessment of 8460 ICD recipients in Denmark that used administrative data was forced to rely on ‘prior diagnosis of ventricular tachycardia or cardiac arrest’ to infer secondary prevention indication; patients without these prior diagnoses were assumed to have primary prevention ICDs.^16^ Applying a similar approach to the CDR (ie, assuming all ICDs are implanted for primary prevention of SCD unless the registry indicates a history of ventricular arrhythmia or cardiac arrest) yields a sensitivity of 64% and a specificity of 73% (Supplemental Table S9). In contrast, our complete registry data predictive model exhibits a sensitivity of 89% and a specificity of 88% in the CDR. In the future, our findings might be used to reliably establish indication for implantation for a greater proportion of study participants, thereby improving statistical power, potentially reducing exposure misclassification, and potentially facilitating more accurate assessment of cost-effectiveness.

Examining the benefits and risks of primary prevention ICD implantation under current guidelines is one such opportunity for research.^8^^,^^26^ Modern primary prevention ICD shock incidence is extremely low; only 0.75% of patients are expected to receive an appropriate therapy in response to an arrhythmia within the first 6 months after implantation.^9^ Half of all ICD patients aged 65 years or older are expected to die or enter hospice care within 5 years of implantation.^27^ Many patients will thus be exposed to the risks and expense of ICD implantation without ever receiving a therapy or any survival benefit from their ICD.^4^^,^^5^^,^^8^^,^^27^ Our model presents an opportunity to use routinely collected health data to study these patients.

Our findings also can be used to strengthen the use of routinely collected health data to monitor health system performance. Several European administrative hospital discharge databases have been validated for tracking ICD implantation rates using the European Heart Rhythm Association’s (EHRA) comprehensive device monitoring system, the EHRA White Book, as a reference standard.^11^ Another recent study examined 585 ICD implantations in Nova Scotia (Canada) and found that administrative data could identify ICD complications and infections with 92% sensitivity and 100% specificity, compared to a reference standard of retrospective chart review.^28^ Our findings might be of use because neither the European nor the Canadian discharge databases document the indication for ICD implantation. Efforts to evaluate disparities in access to ICD implantation based on sex, race, socioeconomic factors, and insurance status could also be strengthened substantially using imputed data on ICD indication.^10^ Our findings thus will make administrative health data far more useful for monitoring complications, assessing disparities in access to primary prevention ICDs, evaluating compliance with clinical guidelines, and informing health policy.

Our study has many strengths. We examined a large cohort of ICD patients using population-based data, suggesting our findings can be generalized to other jurisdictions. The 16 year study interval encompasses the publication of current guidelines for ICD implantation and includes modern ICD technological developments such as antitachycardia pacing.^9^^,^^29^ We selected input variables that are routinely available in cardiac device registries and administrative health data so that our predictive model could be applied by researchers and administrators to other datasets. We examined the predictive performance of models based on registry data alone, administrative data alone, and both registry and administrative data, in an effort to make our findings relevant in a variety of contexts. We used 200 bootstraps to correct for optimism in our assessment of model performance. We developed predictive models that exhibited excellent predictive performance, and we identified several highly predictive or deterministically predictive variables that might be useful for future research.^11^^,^^15^ To our knowledge, this model is the first to be validated for predicting ICD indication in cardiac device registry and administrative health databases.

Our study also has limitations. New York Heart Association classification and left ventricle ejection fraction were not documented in the CDR, reducing model performance. As we relied on the Hawkins et al. data^14^ to validate our prediction models for a subset of CSBC registry patients, individuals for whom the reference standard indicated an “indeterminate indication” were excluded from analysis. Our study relies on the unverifiable assumption that ICD indication was missing at random. Our predictive models may not perform well if applied to datasets that do not include strong predictors of secondary prevention ICD (eg, history of cardiac arrest and ventricular arrhythmias, length of hospital stay, and urgency of implantation), but the information we provide on the predictive strength of these variables will inform efforts to derive or validate models based on the available predictors.

## Conclusions

Variables that are commonly available in cardiac device registries and administrative health data can be used to predict ICD indication. The predictive models we developed could be applied to routinely collected health data to study the real-world uptake, benefits, risks, and cost-effectiveness of ICDs.
