# Supplementary material for: Distinguishing Primary Prevention From Secondary Prevention Implantable Cardioverter Defibrillators Using Administrative Health and Cardiac Device Registry Data
Source: CJC Open. 2024 Mar 7;6(7):876–83. doi: 10.1016/j.cjco.2024.02.003 (PMC11252512; doi:10.1016/j.cjco.2024.02.003)
Supplement: Supplemental appendix [file mmc1.pdf]

**Distinguishing primary and secondary prevention**

**implantable cardioverter defibrillators using administrative health and cardiac device registry data**

## Supplementary Material

## Table of contents


| Description | Page |
|---|---|
| Supplemental Table S1. Data sources | 2 |
| Supplemental Table S2. Candidate variables to predict ICD indication using administrative health data | 3 |
| Supplemental Table S3: Diagnostic codes used to identify comorbidities in administrative health data | 4-5 |
| Supplemental Table S4: Registry variables predictive of secondary prevention ICDs in the CDR | 6-7 |
| Supplemental Table S5: Registry variables predictive of secondary prevention ICDs in HEARTis | 8 |
| Supplemental Table S6: Administrative health data variables predictive of secondary prevention ICDs in the CDR and HEARTis | 9-12 |
| Supplemental Table S7: Combined registry and administrative health data variables predictive of secondary prevention ICDs in CDR | 13-15 |
| Supplemental Table S8: Combined registry and administrative health data variables predictive of secondary prevention ICDs in HEARTis | 16-18 |
| Supplemental Table S9: Performance of rule-based ICD prediction methods | 19 |
| Supplemental Table S10: STROBE checklist | 20 |
| References | 22 |

**Supplemental Table S1: Data sources**

| Health Data | Source |
|---|---|
| ICD implantation data | Cardiac Services BC (CSBC) cardiac device registries (CDR and HEARTis) |
| Medical Services Plan registration | Consolidation File |
| Hospitalization records | Discharge Abstract Database (DAD) |
| Physician services | BC Medical Service Plan (MSP) |
| Prescription drug fill data | PharmaNet |
| Residential neighbourhood-level household income | Income Band database |
| Date and cause of death | Vital Statistics database |

- **Cardiac Services BC**: Cardiac Services BC. Vancouver, BC: Provincial Health Services Authority, 2021. http://www.cardiacbc.ca/
    - Cardiac Devices registry (1995-2013); HEARTis registry (2013-2019)
- **Consolidation File**: British Columbia Ministry of Health [creator] (2020): Consolidation File (MSP Registration & Premium Billing). V2. Population Data BC [publisher]. Data Extract. MOH (2020). http://www.popdata.bc.ca/data
- **Discharge Abstract Database**: Canadian Institute for Health Information [creator] (2020): Discharge Abstract Database (Hospital Separations). V2. Population Data BC [publisher]. Data Extract. MOH (2020). http://www.popdata.bc.ca/data
- **Medical Services Plan**: British Columbia Ministry of Health [creator] (2020): Medical Services Plan (MSP) Payment Information File. Population Data BC [publisher]. Data Extract. MOH (2020). http://www.popdata.bc.ca/data
- **PharmaNet**: British Columbia Ministry of Health [creator] (2020): PharmaNet. V2. Population Data BC [publisher]. Data Extract. Data Stewardship Committee (2020). http://www.popdata.bc.ca/data
- **Income Band**: Statistics Canada [creator]: Statistics Canada Income Band Data. Catalogue Number: 13C0016. V2. Population Data BC [publisher]. Data Extract. Population Data BC (2020). http://www.popdata.bc.ca/data
- **Vital Statistics**: British Columbia Ministry of Health [creator] (2020): Vital Events Deaths. V2. Population Data BC [publisher]. Data Extract. MOH (2020). http://www.popdata.bc.ca/data

All inferences, opinions and conclusions drawn in this manuscript are those of the authors and do not reflect the opinions or policies of the Data Stewards. Access to data provided by the Data Stewards is subject to approval but can be requested for research projects through the Data Stewards or their designated service providers.

**Supplemental Table S2: Candidate variables to predict ICD indication using administrative health data**

| Variables | Definition |
|---|---|
| **Demographic** | |
| Age | Age in years; continuous. |
| Male sex | Sex recorded as male (reference: sex recorded as female, unknown, or missing). |
| Residential neighbourhood household income quintile | Residential neighbourhood income quintile generated by PopData using census data and patient residential postal code (1 = lowest income quintile*, 5 = highest income quintile; ordinal). |
| **Health services use** | |
| Total days in hospital in the past Year | Continuous variable for the number of days in hospital in the year before implantation date. |
| Physician visits in the past year | Number of physician/clinic visits in the year before implantation date; 0 visits*, ≤ 1 per month (1-12 visits); ≤ 1 per week (13-52 visits); > 1 per week (>52 visits) |
| Recent hospitalization for myocardial infarction | Discharge date of hospitalization with Most Responsible Diagnosis corresponding to myocardial infarction (see diagnostic codes in Appendix **Supplemental Table S3**) ≤6 weeks prior to implantation date. |
| Index visit deemed 'urgent' instead of 'elective' | DAD's ADMIT variable is 'U' (urgent) instead of 'L' (elective) or any other response. |
| Prolonged length of stay | Index visit length of stay ≥3 days. |
| Selected index visit Most Responsible Diagnoses | Identified within the Discharge Abstract Database entry for the index visit for ICD implantation; see **Supplemental Table S3** for specific diagnostic codes. Specific MRDs of interest include cardiac arrest; ventricular tachycardia and fibrillation; other arrhythmias; syncope; pacemaker; presence of ICD; heart failure; myocardial infarction OR unstable angina; chronic ischemic heart disease. |
| **Comorbidities** | |
| CCI in the past year | Dichotomized variable for CCI ≥2 (yes/no). |
| Selected comorbidities | Comorbidities were deemed to be present if the associated diagnostic codes were found in ≥1 hospitalization or ≥2 clinic visits in the year before index date.[1] Specific comorbidities of interest and their corresponding diagnostic codes are listed in **Supplemental Table S3**. |
| **Medications** | |
| Number of active prescription medications at index date | Number of active prescription medications at index date (0*, 1, ≥2 distinct medications). |
| Selected medication cases | Medications were deemed to be present if active at implantation date, based on prescription fill date and days supplied. |

**Legend**. Potential health variables obtained from administrative health datasets that may be predictive of ICD indication when it is missing from cardiac registry datasets. * indicates the referent. DAD, Discharge Abstract Database; CCI, Charlson Comorbidity Index; MRD, Most responsible diagnosis.

**Supplemental Table S3: Diagnostic codes used to identify comorbidities in administrative health data**

| Condition | Codes |
|---|---|
| **Myocardial infarction** | ICD9: 410, 412; ICD10: I21, I22, I252 |
| **Congestive heart failure** | ICD9: 39891, 402, 404, 425, 428; ICD10: I43, I50, I099, I110, I130, I132, I255, I420, I425, I426, I427, I428, I429, P290 |
| **Peripheral vascular disease** | ICD9: 0930, 437, 440, 441, 443, 4471, 5571, 5579, V434; ICD10: I70, I71, I731, I738, I739, I771, I790, I792, K551, K558, K559, Z958, Z959 |
| **Cerebrovascular disease** | ICD9: 36234, 430-438; ICD10: G45, G46, I60-I69, H340 |
| **Dementia** | ICD9: 290, 2941, 3312; ICD10: F00-F03, G30, F051, G311 |
| **Chronic obstructive pulmonary disease** | ICD9: 4168, 4169, 490-496, 500-505, 5064, 5081, 5088; ICD10: J40-J47, J60-J67, I278, I279, J684, J701, J703 |
| **Rheumatic disease** | ICD9: 4465, 7100-7104, 7140-7142, 7148, 725; ICD10: M05, M32-M34, M06, M315, M351, M353, M360 |
| **Peptic ulcer disease** | ICD9: 531-534; ICD10: K25-K28 |
| **Mild liver disease** | ICD9: 07022, 07023, 07032, 07033, 07044, 07054, 0706, 0709, 570, 571, 5733, 5734, 5738, 5739, V427; ICD10: B18, K73, K74, K700-K703, K709, K717, K713-K715, K760, K762- K764, K768, K769, Z944 |
| **Diabetes without complications** | ICD9: 2500-2503, 2508, 2509; ICD10: E100, E101, E106, E108, E109, E110, E111, E116, E118, E119, E120, E121, E126, E128, E129, E130, E131, E136, E138, E139, E140, E141, E146, E148, E149 |
| **Diabetes with complications** | ICD9: 2504-2507; ICD10: E102- E105, E107, E112-E115, E117, E122-E125, E127, E132-E135, E137, E142-E145, E147 |
| **Paraplegia and hemiplegia** | ICD9: 3341, 342, 343, 3440-3446, 3449; ICD10: G81, G82, G041, G114, G801, G802, G830-G834, G839 |
| **Renal disease** | ICD9: 403, 404, 582, 5830, 5831, 5832, 5834, 5836, 5837, 585, 586, 5880, V420, V451, V56; ICD10: N18, N19, N052-N057, N250, I120, I131, N032-N037, Z490-Z492, Z940, Z992 |
| **Cancer** | ICD9: 140-165, 170-172, 174-176, 179-195, 200- 208, 2386; ICD10: C00-C26, C30-C34, C37-C41, C43, C45-C58, C60-C76, C81-C85, C88, C90-C97 |
| **Moderate or severe liver disease** | ICD9: 4560-4562, 5722-5724, 5728; ICD10: K704, K711, K721, K729, K765-K767, I850, I859, I864, I982 |
| **Metastatic carcinoma** | ICD9: 196-199; ICD10: C77-C80 |
| **HIV** | ICD9: 042; ICD10: B20-B24 |
| **Syncope** | ICD9: 7802; ICD10: R55 |
| **Atrial fibrillation and flutter** | ICD9: 427; ICD10: I48 |
| **Other arrhythmias** | ICD9: 426, 427, 74686, 7850; ICD10: I44-I47, I49, R00 |
| **Presence of AICD** | ICD9: 99604, V4502, V5332; ICD10: Z9501, Z9502, Z4501, Z4502 |
| **Seizure disorders** | ICD9: 345, 78033; ICD10: G40, R5680 |
| **Obstructive sleep apnea and other sleep disorders** | ICD9: 307, 327, 3270, 32711, 32712, 3272, 32720, 32721, 32723, 32724, 32726, 32727, 32729, 3273, 32730, 32731, 32732, 32733, 32734, 32735, 32736, 32737, 32739, 32742, 32743, 3275, 32752, 32753, 32759, 3278, 7805, 78050, 78051, 78053, 78055-78059, V694; ICD10: F51, G47 |
| **Traumatic brain injury** | ICD9: 310, 80009, 80019, 80029, 80039, 80049, 80059, 80069, 80079, 80089, 80099, 80109, 80119, 80129, 80139, 80149, 80159, 80169, 80179, 80189, 80199, 80309, 80319, 80329, 80339, 80349, 80359, 80369, 80379, 80389, 80399, 80409, 80419, 80429, 80439, 80449, 80459, 80469, 80479, 80489, 80499, 850, 8500, 85011, 85012, 8502, 8503, 8504, 8505, 8509, 85109, 85119, 85129, 85139, 85149, 85159, 85169, 85179, 85189, 85199, 85209, |

| | |
|---|---|
| | 85219, 85229, 85239, 85249, 85259, 85309, 85319, 85409, 85419, V1552, V8001; ICD10: F072, S06 |
| **Psychiatric disorders** | ICD9: 295, 296, 297, 298, 299, 300, 301, 306, 307, 308, 309, 310, 311, 312, 313, 314, 315, 316, 317, 318, 319; ICD10: F04, F05, F06, F07, F08, F09, F2, F3, F4, F5, F6, F7, F8, F9 |
| **Alcohol misuse** | ICD9: 291, 303, 305, 3575, 4255, 5353, 53530, 53531, 571, 76071, 7903, 9773, 980, E860, V113; ICD10: F10, R780, T51, X45, X65, Y15, Y90, Y91, Z502, Z714, Z721, Z8640 |
| **Other substance misuse** | ICD9: 292, 304, 305, 76073, 76075, 9650, 96501, 96502, 96509, 967, 9670, 9671, 9676, 9678, 9679, 9696, 96972, 9701, 97081, E8500, E8501, E8502, E851, E852, E8520, E8525, E8528, E8529, E8541, E9350, E9351, E9352, E937, E9370, E9371, E9376, E9378, E9379, E9396, E9401, E9501, E9502, E9801, E9802; ICD10: F11, F12, F13, F14, F15, F16, F17, F18, F19 |
| **Chronic ischemic heart disease** | ICD9: 414, 4140, 41400, 41401, 41402, 41403, 41404, 41405, 41406, 41407, 4143, 4144, 4148, 4149; ICD10: I25 |
| **Hypertension** | ICD9: 36211, 401, 402, 403, 404, 405, 6420, 64200, 64201, 64202, 64203, 64204, 6421, 64210, 64211, 64212, 64213, 64214, 6422, 64220, 64221, 64222, 64223, 64224, 6427, 64270, 64271, 64272, 64273, 64274; ICD10: I10, I11, I12, I13, I14, I15, O10, O11 |
| **Pacemaker** | ICD9: V4501, V5331; ICD10: Z4500, Z9500, z9502, z4502 |
| **Cardiac arrest** | ICD9: 427, 77985, 798, V1253; ICD10: I46, R96 |
| **Ventricular tachycardia and fibrillation** | ICD9: 427; ICD10: I472, I490 |
| **Unstable angina** | ICD9: None; ICD10: I200 |

*Legend.* ICD in this table refers to the International Classification of Diseases.

**Supplemental Table S4: Registry variables predictive of secondary prevention ICDs in CDR**

| Variable | ICD implantations with feature, count (%) | Odds ratio (95%CI), p-value | Estimate |
|---|---|---|---|
| Intercept* | 3008 (100) | 0.06 (0.02, 0.16), p<0.001 | -2.8756 |
| History of cardiac arrest, VF, or Brugada syndrome* | 570 (19) | 2440.86 (768.41, 7753.38), p<0.001 | 7.8001 |
| History of VT* | 1311 (44) | 46.25 (27.13, 78.84), p<0.001 | 3.8340 |
| History of syncope* | 669 (22) | 9.89 (4.59, 21.30), p<0.001 | 2.2917 |
| Current medication: Alpha blockers | 25 (1) | 2.49 (0.45, 13.73), p=0.294 | 0.9130 |
| Clinical presentation: Syncope | 544 (18) | 2.13 (0.90, 5.05), p=0.086 | 0.7564 |
| Primary or final diagnosis of ARVD | 11 (0) | 2.08 (0.31, 13.89), p=0.449 | 0.7329 |
| Clinical presentation: Missing* | 565 (19) | 2.02 (1.34, 3.03), p=0.001 | 0.7008 |
| Tachyarrhythmia description: Missing | 226 (8) | 1.75 (0.88, 3.51), p=0.112 | 0.5623 |
| History of stroke | 137 (5) | 1.70 (0.81, 3.53), p=0.158 | 0.5283 |
| History of MI ≤6 weeks prior | 102 (3) | 1.59 (0.71, 3.54), p=0.256 | 0.4639 |
| History of ischemic heart failure | 333 (11) | 1.56 (0.80, 3.03), p=0.19 | 0.4448 |
| History of heart block | 76 (3) | 1.51 (0.61, 3.76), p=0.377 | 0.4113 |
| History of nonischemic heart failure | 151 (5) | 1.29 (0.47, 3.53), p=0.623 | 0.2529 |
| Diagnosis of long QT syndrome | 46 (2) | 1.27 (0.41, 3.92), p=0.682 | 0.2361 |
| History of hypertension | 793 (26) | 1.20 (0.83, 1.72), p=0.331 | 0.1791 |
| Previous congenital heart surgery | 11 (0) | 1.20 (0.14, 10.26), p=0.868 | 0.1814 |
| Previous stent/angioplasty | 467 (16) | 1.17 (0.76, 1.80), p=0.465 | 0.1598 |
| History of ischemic heart disease | 1505 (50) | 1.15 (0.66, 1.99), p=0.63 | 0.1357 |
| History of valvular heart disease | 376 (13) | 1.14 (0.74, 1.77), p=0.545 | 0.1342 |
| History of heart failure | 1648 (55) | 1.11 (0.67, 1.83), p=0.695 | 0.1006 |
| History of peripheral vascular disease | 121 (4) | 1.10 (0.57, 2.12), p=0.779 | 0.0939 |
| History of MI >6 weeks prior | 800 (27) | 1.09 (0.73, 1.62), p=0.671 | 0.0860 |
| Current medication: Class III antiarrhythmics | 561 (19) | 1.06 (0.77, 1.46), p=0.704 | 0.0616 |
| ICD models C174AWK, D234TRK, or D354TRG | 556 (18) | 1.03 (0.65, 1.62), p=0.902 | 0.0285 |
| Current medication: Anti-hyperlipidemics | 1477 (49) | 1.02 (0.73, 1.41), p=0.923 | 0.0161 |
| Age in years | Non-binary variable | 1.01 (0.99, 1.02), p=0.416 | 0.0051 |
| Previous acquired heart surgery | 606 (20) | 1.00 (0.69, 1.46), p=0.986 | 0.0034 |
| Current medication: ACE inhibitors | 1964 (65) | 0.99 (0.73, 1.35), p=0.953 | -0.0093 |
| Current medication: Diuretics | 1449 (48) | 0.95 (0.70, 1.30), p=0.754 | -0.0492 |
| Male sex | 2465 (82) | 0.95 (0.65, 1.38), p=0.795 | -0.0497 |
| Current medications: None | 106 (4) | 0.93 (0.41, 2.10), p=0.853 | -0.0776 |
| History of diabetes | 481 (16) | 0.90 (0.60, 1.37), p=0.636 | -0.0999 |
| Current medication: Class I antiarrhythmics | 16 (1) | 0.90 (0.18, 4.42), p=0.901 | -0.1013 |
| Current medication: Antiplatelet agents | 1501 (50) | 0.89 (0.66, 1.19), p=0.42 | -0.1201 |
| Current medication: Anticoagulants | 943 (31) | 0.86 (0.63, 1.18), p=0.342 | -0.1518 |
| Clinical presentation: Asymptomatic | 377 (13) | 0.84 (0.54, 1.31), p=0.449 | -0.1705 |

| | | | |
|---|---|---|---|
| Current medication: Digoxin | 440 (15) | 0.84 (0.54, 1.30), p=0.431 | -0.1740 |
| Current medication: Class IV antiarrhythmics | 198 (7) | 0.84 (0.47, 1.49), p=0.549 | -0.1745 |
| Current medication: Nitrates | 290 (10) | 0.83 (0.54, 1.28), p=0.405 | -0.1829 |
| No known history of cardiovascular disease | 162 (5) | 0.76 (0.39, 1.45), p=0.401 | -0.2793 |
| Clinical presentation: Heart failure | 978 (33) | 0.72 (0.45, 1.15), p=0.171 | -0.3243 |
| History of coronary artery disease | 1713 (57) | 0.69 (0.35, 1.33), p=0.267 | -0.3766 |
| ICD indication reported to be primary | 783 (26) | 0.61 (0.35, 1.05), p=0.076 | -0.4937 |
| Current medication: Class II antiarrhythmics* | 2281 (76) | 0.60 (0.43, 0.83), p=0.002 | -0.5144 |
| Implantation 'elective' instead of 'urgent'/'emergency'* | 2052 (68) | 0.57 (0.42, 0.76), p<0.001 | -0.5632 |
| History of cardiomyopathy* | 871 (29) | 0.54 (0.34, 0.84), p=0.006 | -0.6243 |
| History of congenital heart disease | 40 (1) | 0.37 (0.12, 1.10), p=0.074 | -1.0025 |
| Tachyarrhythmia description: No tachyarrhythmia* | 639 (21) | 0.09 (0.05, 0.17), p<0.001 | -2.3818 |

*Legend*. Table presenting the strength of association between specific clinical registry variables from CDR and ICD indication. Main finding is that predictors of secondary-prevention ICD are plausible. CI, confidence interval; * indicates p<0.05; VT, ventricular tachycardia; MI, myocardial infarction; ARVD, arrhythmogenic right ventricular dysplasia; ACE, angiotensin-converting enzyme.

**Supplemental Table S5: Registry variables predictive of secondary prevention in HEARTis**

| Variable | ICD implantations with feature, count (%) | Odds ratio (95% CI), p-value | Estimate |
|---|---|---|---|
| Intercept | 2928 (100) | 0.77 (0.37, 1.61), p=0.486 | -0.2614 |
| History of cardiac arrest, VF, or Brugada syndrome | 475 (16) | 512,496,911.17 (0.00, Inf), p=0.96 | 20.0548 |
| History of VT* | 850 (29) | 6.39 (4.85, 8.43), p<0.001 | 1.8554 |
| Left ventricle ejection fraction ≥35% (ref: missing)* | 811 (28) | 3.96 (2.62, 5.98), p<0.001 | 1.3761 |
| History of channelopathy* | 57 (2) | 3.49 (1.23, 9.88), p=0.019 | 1.2492 |
| History of syncope* | 518 (18) | 3.12 (2.27, 4.28), p<0.001 | 1.1370 |
| Current medication: Type I or Type III antiarrhythmics* | 497 (17) | 2.40 (1.72, 3.37), p<0.001 | 0.8771 |
| Dual chamber ICD (ref: single chamber)* | 1155 (39) | 1.77 (1.30, 2.41), p<0.001 | 0.5716 |
| Left ventricle ejection fraction <= 34 (ref: missing)* | 1214 (41) | 1.64 (1.03, 2.62), p=0.037 | 0.4974 |
| History of coronary artery disease | 668 (23) | 1.34 (0.98, 1.84), p=0.065 | 0.2944 |
| Age in years | Non-binary variable | 1.01 (1.00, 1.02), p=0.262 | 0.0062 |
| Male sex | 2329 (80) | 1.00 (0.72, 1.39), p=0.988 | 0.0025 |
| NYHA Class I (ref: missing) | 465 (16) | 0.96 (0.65, 1.40), p=0.815 | -0.0453 |
| NYHA Class IV (ref: missing) | 35 (1) | 0.92 (0.33, 2.52), p=0.867 | -0.0866 |
| History of heart failure | 1850 (63) | 0.83 (0.55, 1.27), p=0.394 | -0.1827 |
| NYHA Class II (ref: missing)* | 703 (24) | 0.65 (0.47, 0.90), p=0.01 | -0.4291 |
| History of atrial tachycardia* | 790 (27) | 0.62 (0.46, 0.83), p=0.001 | -0.4844 |
| NYHA Class III (ref: missing)* | 302 (10) | 0.55 (0.35, 0.86), p=0.009 | -0.6017 |
| CRT ICD (ref: single chamber)* | 900 (31) | 0.44 (0.31, 0.63), p<0.001 | -0.8171 |
| Implantation 'elective' instead of 'urgent'/'emergency'* | 1747 (60) | 0.06 (0.05, 0.08), p<0.001 | -2.8111 |

*Legend*. Table presenting the strength of association between specific clinical registry variables used in HEARTis and ICD indication. * indicates p<0.05; VT, ventricular tachycardia; VF, ventricular fibrillation; NYHA, New York Heart Association; CRT, cardiac resynchronization therapy.

**Supplemental Table S6: Administrative health data variables predictive of secondary prevention ICDs in the CDR and HEARTis**

| Variable | ICDs with feature in CDR, count (%) | ICDs with feature in HEARTis, count (%) | CDR odds ratio (95%CI), p-value | HEARTis odds ratio (95%CI), p-value |
|---|---|---|---|---|
| Intercept | 3008 (100) | 2928 (100) | 0.62 (0.34, 1.13), p=0.117 | 0.22 (0.09, 0.51), p<0.001* |
| Implantation 'urgent/emergency' instead of 'elective' | 1081 (36) | 1445 (49) | 1.43 (1.08, 1.89), p=0.014* | 4.35 (2.92, 6.47), p<0.001* |
| Index length of stay 3 or more days | 1116 (37) | 1437 (49) | 1.51 (1.14, 2.00), p=0.004* | 2.92 (1.99, 4.30), p<0.001* |
| Hospitalization for myocardial infarction within 6 weeks of index | 101 (3) | 158 (5) | 1.20 (0.68, 2.12), p=0.527 | 1.55 (0.78, 3.07), p=0.214 |
| Neighbourhood income quintile (ordinal, 1 low 5 high) | Non-binary variable | Non-binary variable | 0.99 (0.93, 1.06), p=0.781 | 1.04 (0.94, 1.14), p=0.438 |
| Number of days spent in hospital year prior to index | Non-binary variable | Non-binary variable | 1.02 (1.01, 1.03), p<0.001* | 1.01 (1.00, 1.02), p=0.274 |
| Age (continuous) | Non-binary variable | Non-binary variable | 1.00 (0.99, 1.01), p=0.812 | 1.01 (1.00, 1.02), p=0.236 |
| Male sex | 2465 (82) | 2324 (79) | 1.09 (0.85, 1.41), p=0.497 | 0.89 (0.63, 1.25), p=0.495 |
| Between 13 and 52 MSP visits in year prior to index (ref: 12 or less) | 2051 (68) | 2058 (70) | 0.88 (0.60, 1.29), p=0.515 | 0.46 (0.27, 0.77), p=0.003* |
| Over 52 MSP visits in year prior to index (ref: 12 or less) | 753 (25) | 629 (21) | 0.53 (0.33, 0.85), p=0.008* | 0.46 (0.24, 0.90), p=0.023* |
| **Comorbidities at index** | | | | |
| Cardiac arrest | 19 (1) | 75 (3) | 9.25 (1.07, 80.37), p=0.044* | 7.60 (0.94, 61.67), p=0.058 |
| Ventricular tachycardia and fibrillation | 683 (23) | 792 (27) | 2.13 (1.63, 2.77), p<0.001* | 7.20 (4.80, 10.81), p<0.001* |
| Syncope | 18 (1) | 38 (1) | 0.91 (0.32, 2.58), p=0.865 | 2.92 (1.23, 6.91), p=0.015* |
| Chronic ischemic heart disease | 391 (13) | 441 (15) | 1.20 (0.84, 1.72), p=0.318 | 1.56 (1.00, 2.45), p=0.052* |
| Myocardial infarction | 36 (1) | 69 (2) | 2.24 (0.79, 6.32), p=0.127 | 1.38 (0.57, 3.36), p=0.48 |
| Atrial fibrillation and flutter | 62 (2) | 110 (4) | 0.60 (0.31, 1.17), p=0.133 | 0.42 (0.21, 0.85), p=0.015* |
| Congestive heart failure | 772 (26) | 922 (31) | 0.41 (0.31, 0.55), p<0.001* | 0.33 (0.23, 0.47), p<0.001* |
| Other arrhythmias | 346 (12) | 121 (4) | 0.98 (0.72, 1.35), p=0.918 | 0.16 (0.09, 0.30), p<0.001* |
| **Comorbidities in the year prior to index** | | | | |

| | | | | |
|---|---|---|---|---|
| Cardiac arrest | 397 (13) | 606 (21) | 11.69 (7.57, 18.04), p<0.001* | 10.02 (6.39, 15.71), p<0.001* |
| Peptic ulcer disease | 30 (1) | 18 (1) | 0.73 (0.29, 1.85), p=0.504 | 7.25 (1.58, 33.35), p=0.011* |
| Pacemaker | N/A | 23 (1) | N/A | 4.18 (0.99, 17.74), p=0.052 |
| Dementia | 19 (1) | 20 (1) | 1.69 (0.46, 6.14), p=0.426 | 3.48 (0.59, 20.33), p=0.167 |
| Paraplegia and hemiplegia | 10 (0) | ≤5 | 0.54 (0.08, 3.47), p=0.515 | 3.10 (0.36, 26.45), p=0.302 |
| Ventricular tachycardia and fibrillation | 1079 (36) | 844 (29) | 1.50 (1.22, 1.84), p<0.001* | 2.78 (2.02, 3.82), p<0.001* |
| Alcohol misuse | 59 (2) | 65 (2) | 0.82 (0.40, 1.69), p=0.585 | 2.40 (0.89, 6.45), p=0.084 |
| Other arrhythmias | 2365 (79) | 2271 (77) | 1.68 (1.29, 2.18), p<0.001* | 2.20 (1.52, 3.18), p<0.001* |
| Hypertension | 972 (32) | 1061 (36) | 1.19 (0.96, 1.48), p=0.119 | 1.48 (1.08, 2.02), p=0.014* |
| Obstructive sleep apnea and other sleep disorders | 65 (2) | 90 (3) | 0.59 (0.29, 1.23), p=0.159 | 1.44 (0.63, 3.32), p=0.39 |
| Binary indicator CCI >= 2 | 1309 (44) | 1329 (45) | 0.84 (0.61, 1.17), p=0.303 | 1.40 (0.89, 2.20), p=0.144 |
| Peripheral vascular disease | 207 (7) | 121 (4) | 0.78 (0.52, 1.17), p=0.227 | 1.28 (0.65, 2.51), p=0.473 |
| Unstable angina | 101 (3) | 37 (1) | 0.67 (0.40, 1.13), p=0.136 | 1.24 (0.34, 4.61), p=0.744 |
| Other substance misuse | 37 (1) | 50 (2) | 0.73 (0.29, 1.84), p=0.507 | 1.15 (0.38, 3.48), p=0.811 |
| Rheumatic disease | 27 (1) | 25 (1) | 1.38 (0.48, 4.00), p=0.551 | 1.06 (0.27, 4.09), p=0.936 |
| Myocardial infarction | 733 (24) | 681 (23) | 1.24 (0.94, 1.64), p=0.132 | 1.00 (0.66, 1.53), p=0.982 |
| Seizure | 18 (1) | 33 (1) | 1.03 (0.27, 3.91), p=0.97 | 0.97 (0.26, 3.72), p=0.97 |
| Atrial fibrillation and flutter | 534 (18) | 594 (20) | 1.27 (0.97, 1.67), p=0.084 | 0.88 (0.60, 1.28), p=0.499 |
| Chronic ischemic heart disease | 1370 (46) | 1415 (48) | 0.95 (0.76, 1.18), p=0.628 | 0.86 (0.63, 1.18), p=0.342 |
| Diabetes with complications | 441 (15) | 598 (20) | 1.21 (0.86, 1.71), p=0.283 | 0.86 (0.54, 1.38), p=0.541 |
| Chronic obstructive pulmonary disease | 324 (11) | 332 (11) | 1.27 (0.90, 1.79), p=0.181 | 0.76 (0.47, 1.21), p=0.243 |
| Cancer | 90 (3) | 114 (4) | 1.54 (0.86, 2.78), p=0.149 | 0.76 (0.35, 1.63), p=0.477 |

| | | | | |
|---|---|---|---|---|
| Syncope | 224 (7) | 283 (10) | 1.60 (1.15, 2.24), p=0.006* | 0.73 (0.48, 1.11), p=0.137 |
| Psychiatric disorders | 323 (11) | 380 (13) | 1.09 (0.79, 1.52), p=0.595 | 0.71 (0.46, 1.08), p=0.107 |
| Renal disease | 231 (8) | 371 (13) | 1.13 (0.76, 1.68), p=0.554 | 0.66 (0.41, 1.05), p=0.081 |
| Cerebrovascular disease | 131 (4) | 130 (4) | 1.22 (0.75, 1.99), p=0.42 | 0.57 (0.29, 1.10), p=0.092 |
| Diabetes without complications | 245 (8) | 170 (6) | 1.39 (0.96, 2.03), p=0.083 | 0.51 (0.28, 0.92), p=0.025* |
| Mild liver disease | 19 (1) | 39 (1) | 0.25 (0.06, 1.03), p=0.055 | 0.47 (0.16, 1.44), p=0.188 |
| Congestive heart failure | 1950 (65) | 1824 (62) | 0.50 (0.40, 0.64), p<0.001* | 0.41 (0.29, 0.57), p<0.001* |
| **Medications active at index date** | | | | |
| ACEi or ARB | 1867 (62) | 1631 (56) | 0.88 (0.67, 1.16), p=0.382 | 1.23 (0.82, 1.85), p=0.326 |
| Antihypertensives | 2363 (79) | 2116 (72) | 0.95 (0.66, 1.38), p=0.797 | 1.05 (0.62, 1.79), p=0.859 |
| Anticoagulants | 660 (22) | 650 (22) | 1.15 (0.88, 1.50), p=0.32 | 1.04 (0.70, 1.54), p=0.862 |
| Anti-arrhythmics | 256 (9) | 123 (4) | 1.20 (0.85, 1.70), p=0.291 | 1.02 (0.54, 1.94), p=0.952 |
| Benzodiazepines | 357 (12) | 243 (8) | 0.84 (0.61, 1.17), p=0.309 | 1.01 (0.61, 1.67), p=0.971 |
| Insulin | 143 (5) | 150 (5) | 0.75 (0.45, 1.25), p=0.273 | 1.01 (0.48, 2.12), p=0.978 |
| Hydralazine | 50 (2) | 46 (2) | 1.51 (0.72, 3.13), p=0.273 | 1.01 (0.31, 3.37), p=0.982 |
| Number of active medications (continuous) | Non-binary variable | Non-binary variable | 0.97 (0.91, 1.04), p=0.387 | 0.99 (0.91, 1.07), p=0.791 |
| Anti-platelets | 461 (15) | 542 (18) | 0.89 (0.65, 1.20), p=0.439 | 0.93 (0.60, 1.44), p=0.734 |
| Calcium channel blockers | 276 (9) | 229 (8) | 1.35 (0.96, 1.90), p=0.083 | 0.92 (0.52, 1.60), p=0.758 |
| Oral hypoglycemics | 463 (15) | 504 (17) | 1.05 (0.76, 1.46), p=0.775 | 0.91 (0.57, 1.44), p=0.69 |
| Statins | 1448 (48) | 1376 (47) | 1.02 (0.80, 1.30), p=0.877 | 0.87 (0.60, 1.26), p=0.462 |
| Nitroglycerin | 306 (10) | 180 (6) | 1.29 (0.92, 1.81), p=0.137 | 0.87 (0.47, 1.61), p=0.655 |
| Beta blockers | 1852 (62) | 1688 (58) | 0.78 (0.59, 1.03), p=0.077 | 0.79 (0.52, 1.19), p=0.261 |
| Loop diuretics | 1094 (36) | 821 (28) | 0.80 (0.62, 1.03), p=0.086 | 0.70 (0.48, 1.03), p=0.072 |

| | | | | |
|---|---|---|---|---|
| MRA | 788 (26) | 830 (28) | 0.61 (0.47, 0.79), p<0.001* | 0.60 (0.41, 0.88), p=0.009* |
| Opioids | 191 (6) | 201 (7) | 1.23 (0.80, 1.91), p=0.346 | 0.56 (0.30, 1.05), p=0.072 |
| Digoxin | 489 (16) | 143 (5) | 0.97 (0.72, 1.29), p=0.821 | 0.54 (0.27, 1.08), p=0.081 |

*Legend.* Table presenting the strength of association between specific administrative variables and ICD indication in the HEARTis and CDR study intervals. CI, confidence interval; * indicates p<0.05; MSP, Medical Services Plan; CCI, Charlson comorbidity index; ACEi, angiotensin-converting enzyme inhibitor; ARB, angiotensin receptor blocker; MRA, Mineralocorticoid receptor antagonist.

**Supplemental Table S7: Combined registry and administrative health data variables predictive of secondary prevention ICDs in CDR**

| Variable | ICD implantations with feature, count (%) | Odds ratio (95%CI), p-value | Estimate |
|---|---|---|---|
| Intercept* | 3008 (100) | 0.04 (0.01, 0.14), p<0.001 | -3.1452 |
| History of cardiac arrest, VF, or Brugada syndrome* | 570 (19) | 2577.09 (747.97, 8879.20), p<0.001 | 7.8544 |
| History of VT* | 1311 (44) | 45.53 (26.53, 78.13), p<0.001 | 3.8184 |
| Clinical presentation: Syncope* | 552 (18) | 6.03 (3.02, 12.03), p<0.001 | 1.797 |
| History of cardiac arrest* | 397 (13) | 5.64 (2.71, 11.73), p<0.001 | 1.7291 |
| Hydralazine* | 50 (2) | 4.75 (1.29, 17.46), p=0.019 | 1.5573 |
| History of syncope* | 753 (25) | 2.94 (1.74, 4.94), p<0.001 | 1.0769 |
| Current medication: Alpha blockers | 25 (1) | 2.44 (0.45, 13.24), p=0.3 | 0.8938 |
| History of cancer | 90 (3) | 2.17 (0.92, 5.09), p=0.076 | 0.7729 |
| History of chronic obstructive pulmonary disease* | 324 (11) | 2.01 (1.22, 3.31), p=0.006 | 0.6972 |
| History of stroke | 137 (5) | 1.99 (0.92, 4.32), p=0.08 | 0.6896 |
| History of seizure | 18 (1) | 1.92 (0.29, 12.58), p=0.496 | 0.6533 |
| Primary or final diagnosis of ARVD | 11 (0) | 1.77 (0.27, 11.79), p=0.554 | 0.5720 |
| Chronic ischemic heart disease at index | 391 (13) | 1.64 (0.96, 2.80), p=0.07 | 0.4950 |
| Clinical presentation: Missing* | 565 (19) | 1.62 (1.07, 2.44), p=0.022 | 0.4823 |
| History of heart block | 76 (3) | 1.62 (0.62, 4.24), p=0.323 | 0.4846 |
| Other arrhythmias at index* | 346 (12) | 1.50 (1.02, 2.20), p=0.039 | 0.4057 |
| History of other arrhythmias | 2365 (79) | 1.46 (0.97, 2.19), p=0.071 | 0.3752 |
| Tachyarrhythmia description: Missing | 226 (8) | 1.44 (0.69, 3.00), p=0.332 | 0.3631 |
| History of MI ≤6 weeks prior | 176 (6) | 1.43 (0.76, 2.70), p=0.272 | 0.3567 |
| History of nonischemic heart failure | 151 (5) | 1.35 (0.48, 3.78), p=0.568 | 0.2999 |
| ICD models C174AWK, D234TRK, or D354TRG | 556 (18) | 1.31 (0.81, 2.12), p=0.272 | 0.2701 |
| Calcium channel blockers | 276 (9) | 1.31 (0.79, 2.19), p=0.301 | 0.2700 |
| Implantation 'urgent/emergency' instead of 'elective' | 1081 (36) | 1.29 (0.83, 2.00), p=0.255 | 0.2540 |
| Beta blockers | 1852 (62) | 1.26 (0.84, 1.88), p=0.262 | 0.2289 |
| History of renal disease | 231 (8) | 1.24 (0.69, 2.24), p=0.47 | 0.2171 |
| History of hypertension | 1405 (47) | 1.23 (0.89, 1.70), p=0.202 | 0.2084 |
| Previous stent/angioplasty | 467 (16) | 1.23 (0.79, 1.91), p=0.368 | 0.2042 |
| History of ischemic heart failure | 1499 (50) | 1.22 (0.88, 1.68), p=0.235 | 0.1967 |
| History of diabetes | 765 (25) | 1.22 (0.76, 1.95), p=0.408 | 0.1978 |
| History of psychiatric disorders | 323 (11) | 1.17 (0.71, 1.93), p=0.545 | 0.1547 |
| Myocardial infarction at index | 36 (1) | 1.16 (0.27, 5.01), p=0.841 | 0.1495 |
| History of valvular heart disease | 376 (13) | 1.15 (0.72, 1.81), p=0.562 | 0.136 |
| Between 13 and 52 MSP visits in year prior to index (ref: 12 or less) | 2051 (68) | 1.13 (0.65, 1.97), p=0.653 | 0.1263 |
| Over 52 MSP visits in year prior to index (ref: 12 or less) | 753 (25) | 1.13 (0.56, 2.30), p=0.726 | 0.1265 |
| History of ischemic heart disease | 1505 (50) | 1.12 (0.63, 1.98), p=0.697 | 0.1137 |
| Diagnosis of long QT syndrome | 46 (2) | 1.12 (0.35, 3.60), p=0.853 | 0.1108 |
| History of rheumatic disease | 27 (1) | 1.08 (0.26, 4.56), p=0.918 | 0.0753 |
| Previous congenital heart surgery | 11 (0) | 1.07 (0.13, 8.68), p=0.951 | 0.0654 |
| History of paraplegia and hemiplegia | 10 (0) | 1.07 (0.09, 12.28), p=0.957 | 0.0672 |

| | | | |
|---|---|---|---|
| Current medication: Anti-hyperlipidemics | 1477 (49) | 1.06 (0.75, 1.51), p=0.722 | 0.0628 |
| Previous acquired heart surgery | 606 (20) | 1.04 (0.70, 1.55), p=0.835 | 0.0419 |
| History of dementia | 19 (1) | 1.02 (0.14, 7.59), p=0.986 | 0.0182 |
| Anticoagulants | 1117 (37) | 1.01 (0.72, 1.43), p=0.946 | 0.0119 |
| Current medication: Digoxin | 616 (20) | 1.01 (0.67, 1.51), p=0.978 | 0.0057 |
| Number of days spent in hospital year prior to index | Non-binary variable | 1.00 (0.99, 1.02), p=0.605 | 0.0032 |
| Age in years | Non-binary variable | 1.00 (0.99, 1.02), p=0.542 | 0.0042 |
| History of MI >6 weeks prior | 1283 (43) | 1.00 (0.68, 1.46), p=0.982 | -0.0045 |
| History of atrial fibrillation and flutter | 534 (18) | 1.00 (0.67, 1.49), p=0.989 | -0.0029 |
| Benzodiazepines | 357 (12) | 1.00 (0.62, 1.62), p=1 | -0.0001 |
| Antihypertensives | 2363 (79) | 1.00 (0.58, 1.73), p=0.988 | 0.0042 |
| Current medication: Class III antiarrhythmics | 561 (19) | 0.99 (0.71, 1.39), p=0.968 | -0.0070 |
| Statins | 1448 (48) | 0.99 (0.68, 1.43), p=0.955 | -0.0106 |
| Insulin | 143 (5) | 0.99 (0.43, 2.24), p=0.976 | -0.0129 |
| Index length of stay 3 or more days | 1116 (37) | 0.98 (0.63, 1.54), p=0.944 | -0.0161 |
| ACEi or ARB | 1867 (62) | 0.98 (0.60, 1.61), p=0.94 | -0.0190 |
| Opioids | 191 (6) | 0.98 (0.51, 1.87), p=0.943 | -0.0236 |
| History of cerebrovascular disease | 131 (4) | 0.96 (0.44, 2.09), p=0.914 | -0.0430 |
| Neighbourhood income quintile (ordinal, 1 low 5 high) | Non-binary variable | 0.95 (0.86, 1.05), p=0.295 | -0.0520 |
| Atrial fibrillation and flutter at index | 62 (2) | 0.95 (0.34, 2.65), p=0.917 | -0.0547 |
| Number of active medications (continuous) | Non-binary variable | 0.94 (0.87, 1.02), p=0.124 | -0.0628 |
| MRA | 788 (26) | 0.93 (0.62, 1.38), p=0.713 | -0.0753 |
| Male sex | 2465 (82) | 0.92 (0.62, 1.38), p=0.692 | -0.0806 |
| Oral hypoglycemics | 463 (15) | 0.92 (0.55, 1.53), p=0.749 | -0.0837 |
| Current medication: Class I antiarrhythmics | 16 (1) | 0.92 (0.17, 4.92), p=0.925 | -0.0803 |
| Clinical presentation: Asymptomatic | 377 (13) | 0.91 (0.58, 1.42), p=0.669 | -0.0979 |
| Current medication: Nitrates | 481 (16) | 0.89 (0.60, 1.31), p=0.545 | -0.1218 |
| Current medication: ACE inhibitors | 2427 (81) | 0.87 (0.52, 1.45), p=0.591 | -0.1398 |
| Current medication: Diuretics | 1698 (56) | 0.83 (0.58, 1.18), p=0.288 | -0.1917 |
| History of heart failure | 2271 (75) | 0.83 (0.55, 1.24), p=0.362 | -0.1884 |
| Current medication: Antiplatelet agents | 1623 (54) | 0.81 (0.60, 1.10), p=0.175 | -0.2123 |
| History of coronary artery disease | 1713 (57) | 0.80 (0.41, 1.58), p=0.519 | -0.2232 |
| ICD indication reported to be primary | 783 (26) | 0.78 (0.44, 1.38), p=0.386 | -0.2541 |
| History of peripheral vascular disease | 302 (10) | 0.76 (0.47, 1.23), p=0.262 | -0.2769 |
| Current medication: Class IV antiarrhythmics | 198 (7) | 0.73 (0.39, 1.37), p=0.327 | -0.3161 |
| No known history of cardiovascular disease | 162 (5) | 0.73 (0.37, 1.43), p=0.361 | -0.3125 |
| History of unstable angina | 101 (3) | 0.72 (0.35, 1.46), p=0.358 | -0.3327 |
| History of obstructive sleep apnea and other sleep disorders | 65 (2) | 0.72 (0.23, 2.25), p=0.569 | -0.3334 |
| Clinical presentation: Heart failure | 1339 (45) | 0.70 (0.49, 1.02), p=0.064 | -0.3506 |
| History of alcohol misuse | 59 (2) | 0.67 (0.20, 2.23), p=0.512 | -0.4038 |
| History of peptic ulcer disease | 30 (1) | 0.66 (0.13, 3.40), p=0.621 | -0.413 |
| Implantation 'elective' instead of 'urgent'/'emergency'* | 2052 (68) | 0.64 (0.47, 0.88), p=0.006 | -0.4455 |
| Current medication: Class II antiarrhythmics* | 2281 (76) | 0.64 (0.46, 0.90), p=0.01 | -0.4397 |
| History of cardiomyopathy* | 871 (29) | 0.64 (0.44, 0.93), p=0.019 | -0.4509 |
| Binary indicator CCI >= 2* | 1309 (44) | 0.62 (0.40, 0.95), p=0.029 | -0.4841 |

| | | | |
|---|---|---|---|
| History of other substance misuse | 37 (1) | 0.55 (0.14, 2.09), p=0.377 | -0.6051 |
| History of congenital heart disease* | 40 (1) | 0.29 (0.09, 0.92), p=0.035 | -1.2241 |
| Tachyarrhythmia description: No tachyarrhythmia* | 639 (21) | 0.09 (0.05, 0.18), p<0.001 | -2.375 |
| History of mild liver disease* | 19 (1) | 0.06 (0.00, 0.83), p=0.036 | -2.8633 |

*Legend.* Table presenting the strength of association between specific administrative and registry variables and ICD indication in the CDR study interval. CI, confidence interval, * indicates p<0.05, VF, ventricular fibrillation; VT, ventricular tachycardia; ARVD, arrhythmogenic right ventricular dysplasia; MSP, Medical Services Plan; MI, myocardial infarction; ACE, angiotensin-converting enzyme; CI, confidence interval; ACEi, angiotensin-converting enzyme inhibitor; ARB, angiotensin receptor blocker; MRA, Mineralocorticoid receptor antagonist CCI, Charlson comorbidity index.

**Supplemental Table S8: Combined registry and administrative health data variables predictive of secondary prevention ICDs in HEARTis**

| Variable | ICD implantations with feature, count (%) | Odds ratio (95%CI), p-value | Estimate |
|---|---|---|---|
| Intercept | 2928 (100) | 0.37 (0.12, 1.08), p=0.07 | -1.0035 |
| History of cardiac arrest, VF, or Brugada syndrome | 475 (16) | 333792441.82 (0.00, Inf), p=0.959 | 19.626 |
| Implantation 'urgent/emergency' instead of 'elective'* | 1445 (49) | 4.45 (2.78, 7.11), p<0.001 | 1.4925 |
| History of VT* | 850 (29) | 3.92 (2.78, 5.53), p<0.001 | 1.3663 |
| Current medication: Type I or Type III antiarrhythmics* | 497 (17) | 2.78 (1.76, 4.40), p<0.001 | 1.0229 |
| Left ventricle ejection fraction >= 35 (ref: missing)* | 811 (28) | 2.58 (1.65, 4.04), p<0.001 | 0.9496 |
| History of channelopathy | 57 (2) | 2.44 (0.70, 8.56), p=0.163 | 0.8927 |
| Index length of stay 3 or more days* | 1437 (49) | 1.62 (1.02, 2.57), p=0.039 | 0.4843 |
| Hospitalization for myocardial infarction within 6 weeks of index | 158 (5) | 1.61 (0.73, 3.57), p=0.24 | 0.4777 |
| History of syncope* | 636 (22) | 1.60 (1.12, 2.29), p=0.01 | 0.4712 |
| Dual chamber ICD (ref: single chamber)* | 1155 (39) | 1.53 (1.04, 2.26), p=0.031 | 0.4270 |
| Left ventricle ejection fraction <= 34 (ref: missing) | 1214 (41) | 1.47 (0.96, 2.25), p=0.074 | 0.3870 |
| NYHA Class IV (ref: missing) | 35 (1) | 1.46 (0.52, 4.14), p=0.472 | 0.3815 |
| History of coronary artery disease | 668 (23) | 1.29 (0.86, 1.93), p=0.219 | 0.2544 |
| NYHA Class I (ref: missing) | 465 (16) | 1.08 (0.67, 1.73), p=0.752 | 0.0761 |
| Neighbourhood income quintile (ordinal, 1 low 5 high) | Non-binary variable | 1.04 (0.93, 1.17), p=0.453 | 0.0427 |
| Age in years* | Non-binary variable | 1.02 (1.00, 1.03), p=0.027 | 0.0163 |
| Number of days spent in hospital year prior to index | Non-binary variable | 1.00 (0.99, 1.01), p=0.544 | -0.0033 |
| Male sex | 2324 (79) | 0.97 (0.65, 1.45), p=0.886 | -0.0294 |
| NYHA Class III (ref: missing) | 302 (10) | 0.90 (0.52, 1.55), p=0.691 | -0.1108 |
| NYHA Class II (ref: missing) | 703 (24) | 0.85 (0.56, 1.28), p=0.431 | -0.1668 |
| History of atrial tachycardia | 982 (34) | 0.61 (0.41, 0.91), p=0.015 | -0.4907 |
| CRT ICD (ref: single chamber)* | 900 (31) | 0.55 (0.34, 0.86), p=0.01 | -0.6050 |
| Over 52 MSP visits in year prior to index (ref: 12 or less) | 629 (21) | 0.47 (0.22, 1.01), p=0.054 | -0.7469 |
| Between 13 and 52 MSP visits in year prior to index (ref: 12 or less)* | 2058 (70) | 0.36 (0.20, 0.65), p=0.001 | -1.0177 |
| Implantation 'elective' instead of 'urgent'/'emergency'* | 1747 (60) | 0.13 (0.09, 0.18), p<0.001 | -2.0692 |
| **Comorbidities at index** | | | |
| Syncope | 38 (1) | 1.60 (0.62, 4.12), p=0.33 | 0.4700 |
| Chronic ischemic heart disease | 441 (15) | 1.36 (0.75, 2.45), p=0.311 | 0.3051 |
| Myocardial infarction | 69 (2) | 0.47 (0.17, 1.33), p=0.156 | -0.7489 |
| Atrial fibrillation and flutter* | 110 (4) | 0.27 (0.11, 0.63), p=0.002 | -1.3215 |
| Congestive heart failure* | 922 (31) | 0.19 (0.12, 0.30), p<0.001 | -1.6389 |
| Other arrhythmias* | 121 (4) | 0.09 (0.05, 0.18), p<0.001 | -2.3839 |
| **Comorbidities in the year prior to index** | | | |

| | | | |
|---|---|---|---|
| Peptic ulcer disease* | 18 (1) | 6.68 (1.29, 34.56), p=0.024 | 1.8993 |
| Dementia | 20 (1) | 6.16 (0.76, 50.30), p=0.09 | 1.8187 |
| Cardiac arrest* | 606 (21) | 4.58 (2.58, 8.12), p<0.001 | 1.5207 |
| Pacemaker | 23 (1) | 3.93 (0.75, 20.58), p=0.106 | 1.3676 |
| Other arrhythmias* | 2271 (78) | 2.29 (1.50, 3.50), p<0.001 | 0.8290 |
| Alcohol misuse | 65 (2) | 2.23 (0.67, 7.33), p=0.189 | 0.7998 |
| Paraplegia and hemiplegia | ≤5 | 1.85 (0.13, 26.76), p=0.65 | 0.6175 |
| Other substance misuse | 50 (2) | 1.55 (0.50, 4.79), p=0.447 | 0.4373 |
| Unstable angina | 37 (1) | 1.47 (0.37, 5.80), p=0.582 | 0.3854 |
| Hypertension | 1061 (36) | 1.35 (0.95, 1.93), p=0.096 | 0.3031 |
| Binary indicator CCI >= 2 | 1329 (45) | 1.24 (0.74, 2.07), p=0.412 | 0.2148 |
| Diabetes with complications | 598 (20) | 1.21 (0.69, 2.12), p=0.497 | 0.1933 |
| Peripheral vascular disease | 121 (4) | 1.14 (0.52, 2.49), p=0.741 | 0.1314 |
| Obstructive sleep apnea and other sleep disorders | 90 (3) | 1.14 (0.44, 2.96), p=0.79 | 0.1297 |
| Myocardial infarction | 681 (23) | 1.07 (0.65, 1.75), p=0.801 | 0.0639 |
| Chronic obstructive pulmonary disease | 332 (11) | 0.99 (0.59, 1.68), p=0.984 | -0.0054 |
| Chronic ischemic heart disease | 1415 (48) | 0.85 (0.59, 1.23), p=0.401 | -0.1575 |
| Psychiatric disorders | 380 (13) | 0.83 (0.51, 1.36), p=0.46 | -0.1866 |
| Renal disease | 371 (13) | 0.60 (0.34, 1.05), p=0.073 | -0.5139 |
| Seizure | 33 (1) | 0.57 (0.11, 3.02), p=0.508 | -0.5634 |
| Cancer | 114 (4) | 0.56 (0.22, 1.42), p=0.225 | -0.5713 |
| Heart failure* | 2375 (81) | 0.55 (0.34, 0.89), p=0.014 | -0.6033 |
| Cerebrovascular disease | 130 (4) | 0.55 (0.26, 1.15), p=0.109 | -0.6064 |
| Rheumatic disease | 25 (1) | 0.53 (0.08, 3.41), p=0.505 | -0.6322 |
| Diabetes without complications* | 170 (6) | 0.46 (0.24, 0.91), p=0.025 | -0.7711 |
| Mild liver disease | 39 (1) | 0.45 (0.11, 1.84), p=0.266 | -0.7990 |
| **Medications active at index date** | | | |
| Hydralazine | 46 (2) | 1.69 (0.34, 8.50), p=0.525 | 0.5243 |
| ACEi or ARB | 1631 (56) | 1.42 (0.89, 2.24), p=0.14 | 0.3474 |
| Anticoagulants | 650 (22) | 1.16 (0.71, 1.89), p=0.558 | 0.1463 |
| Oral hypoglycemics | 504 (17) | 1.06 (0.62, 1.82), p=0.831 | 0.0587 |
| Calcium channel blockers | 229 (8) | 1.06 (0.56, 2.02), p=0.862 | 0.0574 |
| Number of active medications (continuous) | Non-binary variable | 1.03 (0.94, 1.13), p=0.565 | 0.0278 |
| Antihypertensives | 2116 (72) | 0.99 (0.54, 1.81), p=0.968 | -0.0125 |
| Beta blockers | 1688 (58) | 0.96 (0.60, 1.55), p=0.878 | -0.0372 |
| Insulin | 150 (5) | 0.90 (0.39, 2.04), p=0.794 | -0.1098 |
| Anti-platelets | 542 (19) | 0.82 (0.49, 1.40), p=0.472 | -0.1937 |
| Statins | 1376 (47) | 0.81 (0.53, 1.24), p=0.327 | -0.2141 |
| Benzodiazepines | 243 (8) | 0.81 (0.44, 1.48), p=0.492 | -0.2101 |
| Anti-arrhythmics | 123 (4) | 0.63 (0.29, 1.35), p=0.232 | -0.4650 |
| Nitroglycerin | 180 (6) | 0.60 (0.29, 1.23), p=0.162 | -0.5118 |
| MRA* | 830 (28) | 0.58 (0.38, 0.91), p=0.017 | -0.5367 |
| Opioids | 201 (7) | 0.56 (0.27, 1.14), p=0.111 | -0.5821 |
| Digoxin | 143 (5) | 0.55 (0.24, 1.24), p=0.148 | -0.6018 |
| Loop diuretics* | 821 (28) | 0.54 (0.34, 0.84), p=0.006 | -0.6213 |

*Legend.* Table presenting the strength of association between specific administrative and registry variables with ICD indication in the HEARTis study interval. CI, confidence interval, * indicates p<0.05, VF, ventricular fibrillation; VT, ventricular tachycardia; NYHA, New York Heart Association; MSP, Medical Services Plan; CCI, Charlson comorbidity index; ACE, angiotensin-

converting enzyme; ACEi, angiotensin-converting enzyme inhibitor; ARB, angiotensin receptor blocker; MRA, Mineralocorticoid receptor antagonist.

**Supplemental Table S9: Performance of rule-based ICD prediction methods**

| Measure of predictive model performance | CDR (2004-2012) | HEARTis (2013-2019) | All data (2004-2019) |
|---|---|---|---|
| Sensitivity | 64% | 85% | 74% |
| Specificity | 73% | 84% | 78% |

Legend. Performance metrics for a simplified model based on methods from a Danish nationwide assessment of ICD use.[2] These models used our cardiac device registry data and distinguish ICD indication with the following rules: secondary prevention ICD if patient has an ICD code for cardiac arrest or ventricular tachycardia/fibrillation at index or in year prior to index, otherwise an ICD was considered to be for primary prevention.

### Supplemental Table S10: STROBE checklist

| | Item No | Recommendation | Page No |
|---|---|---|---|
| **Title and abstract** | 1 | (*a*) Indicate the study's design with a commonly used term in the title or the abstract | 1, 3 |
| | | (*b*) Provide in the abstract an informative and balanced summary of what was done and what was found | 3 |
| **Introduction** | | | |
| Background/rationale | 2 | Explain the scientific background and rationale for the investigation being reported | 4,5 |
| Objectives | 3 | State specific objectives, including any prespecified hypotheses | 5 |
| **Methods** | | | |
| Study design | 4 | Present key elements of study design early in the paper | 6-8 |
| Setting | 5 | Describe the setting, locations, and relevant dates, including periods of recruitment, exposure, follow-up, and data collection | 5 |
| Participants | 6 | (*a*) *Cohort study*—Give the eligibility criteria, and the sources and methods of selection of participants. Describe methods of follow-up<br>*Case-control study*—Give the eligibility criteria, and the sources and methods of case ascertainment and control selection. Give the rationale for the choice of cases and controls<br>*Cross-sectional study*—Give the eligibility criteria, and the sources and methods of selection of participants | 6,7 |
| | | (*b*) *Cohort study*—For matched studies, give matching criteria and number of exposed and unexposed<br>*Case-control study*—For matched studies, give matching criteria and the number of controls per case | N/A |
| Variables | 7 | Clearly define all outcomes, exposures, predictors, potential confounders, and effect modifiers. Give diagnostic criteria, if applicable | 7,8 Supplemental Tables S2-S3 |
| Data sources/ measurement | 8* | For each variable of interest, give sources of data and details of methods of assessment (measurement). Describe comparability of assessment methods if there is more than one group | 7,8, Supplemental Table S1 & S3 |
| Bias | 9 | Describe any efforts to address potential sources of bias | 8 |
| Study size | 10 | Explain how the study size was arrived at | 6,7, Fig. 1 |
| Quantitative variables | 11 | Explain how quantitative variables were handled in the analyses. If applicable, describe which groupings were chosen and why | 7,8, Supplemental Tables S2-S3 |
| Statistical methods | 12 | (*a*) Describe all statistical methods, including those used to control for confounding | 7,8 |
| | | (*b*) Describe any methods used to examine subgroups and interactions | N/A |
| | | (*c*) Explain how missing data were addressed | 7,8 |
| | | (*d*) *Cohort study*—If applicable, explain how loss to follow-up was addressed<br>*Case-control study*—If applicable, explain how matching of cases and controls was addressed<br>*Cross-sectional study*—If applicable, describe analytical methods taking account of sampling strategy | N/A |
| | | (*e*) Describe any sensitivity analyses | Supplemental Tables S6-S8 |

## Supplemental Table S10: STROBE checklist (continued)

| | | | |
|---|---|---|---|
| **Results** | | | |
| Participants | 13* | (a) Report numbers of individuals at each stage of study—eg numbers potentially eligible, examined for eligibility, confirmed eligible, included in the study, completing follow-up, and analysed | 9, Fig. 1 |
| | | (b) Give reasons for non-participation at each stage | Fig. 1 |
| | | (c) Consider use of a flow diagram | Fig.1 |
| Descriptive data | 14* | (a) Give characteristics of study participants (eg demographic, clinical, social) and information on exposures and potential confounders | 9, Table 1 |
| | | (b) Indicate number of participants with missing data for each variable of interest | Fig. 2 |
| | | (c) *Cohort study*—Summarise follow-up time (eg, average and total amount) | N/A |
| Outcome data | 15* | *Cohort study*—Report numbers of outcome events or summary measures over time | 8,9, Fig. 2 |
| | | *Case-control study*—Report numbers in each exposure category, or summary measures of exposure | N/A |
| | | *Cross-sectional study*—Report numbers of outcome events or summary measures | N/A |
| Main results | 16 | (*a*) Give unadjusted estimates and, if applicable, confounder-adjusted estimates and their precision (eg, 95% confidence interval). Make clear which confounders were adjusted for and why they were included | 9-10, Table 2, Supplemental Tables S4-S8 |
| | | (*b*) Report category boundaries when continuous variables were categorized | N/A |
| | | (*c*) If relevant, consider translating estimates of relative risk into absolute risk for a meaningful time period | N/A |
| Other analyses | 17 | Report other analyses done—eg analyses of subgroups and interactions, and sensitivity analyses | 10, Supplemental Tables S4-S8 |
| **Discussion** | | | |
| Key results | 18 | Summarise key results with reference to study objectives | 11 |
| Limitations | 19 | Discuss limitations of the study, taking into account sources of potential bias or imprecision. Discuss both direction and magnitude of any potential bias | 14 |
| Interpretation | 20 | Give a cautious overall interpretation of results considering objectives, limitations, multiplicity of analyses, results from similar studies, and other relevant evidence | 14 |
| Generalisability | 21 | Discuss the generalisability (external validity) of the study results | 11-13 |
| **Other information** | | | |
| Funding | 22 | Give the source of funding and the role of the funders for the present study and, if applicable, for the original study on which the present article is based | 1 |

**References**


[1] Quan H, Sundararajan V, Halfon P, Fong A, Burnand B, Luthi JC, Saunders LD, Beck CA, Feasby TE, Ghali WA. Coding algorithms for defining comorbidities in ICD-9-CM and ICD-10 administrative data. Med Care. 2005 Nov;43(11):1130-9. doi: 10.1097/01.mlr.0000182534.19832.83. PMID: 16224307.

[2] Schmidt M, Pedersen SB, Farkas DK, Hjortshøj SP, Bøtker HE, Nielsen JC, Sørensen HT. Thirteen-year nationwide trends in use of implantable cardioverter-defibrillators and subsequent long-term survival. Heart Rhythm. 2015 Sep;12(9):2018-27. doi: 10.1016/j.hrthm.2015.04.040. Epub 2015 Apr 30. PMID: 25937527.
